# Complete genome sequence of plasmid-bearing aerotolerant *Campylobacter jejuni* strain S2-20 isolated from retail chicken meat

**DOI:** 10.1128/mra.01141-23

**Published:** 2024-03-14

**Authors:** Anand B. Karki, Elise Delaborte, Mohamed K. Fakhr

**Affiliations:** 1 Department of Biological Science, The University of Tulsa, Tulsa, Oklahoma, USA; 2 Department of Biological Sciences, Sam Houston State University, Huntsville, Texas, USA; University of Maryland School of Medicine, Baltimore, Maryland, USA

**Keywords:** *Campylobacter*, aerotolerance, retail meat, genome sequence, plasmids, oxidative stress

## Abstract

Complete genome sequencing of aerotolerant *Campylobacter jejuni* strain S2-20 revealed the presence of a chromosome of 1,695,449 bp and a plasmid of 49,741 bp that contains predicted antimicrobial resistance and type IV secretion system genes. The chromosome harbored several putative oxidative stress genes with potential roles in aerotolerance.

## ANNOUNCEMENT

*Campylobacter jejuni* remains one of the leading causes of foodborne illness in the United States. *Campylobacter* is microaerophilic; however, in recent years, a high prevalence of aerotolerant *Campylobacter* in retail meat products has been reported (1–6). Aerotolerancy (surviving up to 12 hours of aerobic incubation) enhances *Campylobacter* survival during retail meat processing and storage (1, 7–9).

Here, we announce the complete genome sequence of aerotolerant *Campylobacter jejuni* strain S2-20 previously isolated in our laboratory from retail chicken meat in 2010 at 42°C on *Campylobacter* Desoxycholate Agar after enrichment in Bolton broth (10). The strain can survive for more than 15 hours of aerobic incubation (1) and was shown to harbor one plasmid (11). The strain was cultured in Mueller Hinton Broth for 24 hours under microaerobic conditions, and DNA was isolated using the DNeasy Blood & Tissue Kit (Qiagen, Valencia, CA). DNA was neither sheared nor size-selected before library preparation. Paired-end sequencing of the Nextera XT library (Illumina, Inc., San Diego, CA) was performed on NovaSeq 6000 platform at the Oklahoma Medical Research Foundation Clinical Genomics Center in Oklahoma City to obtain 69,155,464 paired-end reads of 151 bp read length. In addition, a DNA library prepared with Ligation Sequencing Kit (SQK-LSK114, Oxford Nanopore Technology, Lexington, MA, USA) enriching DNA fragments of >3 kb, with long fragment buffer, was sequenced in FLO-MIN-114 flow cell (ONT, Lexington, MA, USA) and base-called (high accuracy base calling, Guppy 6.5.7) on Mk1C platform (MinION Mk1C_23.04.8) to obtain 1,484,094-long reads with N50 of 5,671 bp. The quality of sequence reads was checked with FastQC_v0.11.9 (https://www.bioinformatics.babraham.ac.uk/projects/fastqc/). Short reads from Illumina were trimmed with trimmomatic_v0.39 (12) using the following parameters (ILLUMINACLIP:adapters.fa:2:30:10_LEADING:3_TRAILING:3_SLIDINGWINDOW:4:15_MINLEN:36), and long sequence reads from ONT were trimmed with default settings by porechop_abi_v0.5.0 (13). Hybrid genome assembly was created with Unicycler_v0.5.0 (14), which produced two circular contigs, and the quality of assembly was checked with Quast_v5.2.0 (15). Genome annotation was carried out with Prokaryotic Genome Annotation Pipeline (PGAP_6.6) (https://www.ncbi.nlm.nih.gov/genome/annotation_prok/) (16) and Bacterial and Viral Bioinformatics Resource Center BV-BRC_3.31.12 (https://www.bv-brc.org/app/Annotation) (17). All software and packages used in this study were used with default parameters unless stated separately.

The genome of *Campylobacter jejuni* S2-20 contained a circular chromosome and one circular plasmid. The sizes of the chromosome and plasmid pCJABK49 were 1,695,449 bp (GC content = 30.54) and 49,741 bp (GC content = 29.44), respectively. Using PGAP annotation, the chromosome contained 1,827 genes, 1,771 coding sequences (CDS), 44 tRNAs, and 9 rRNAs. Using the Virulence Factor Database (http://www.mgc.ac.cn/VFs/) (18), the chromosome contained 95 putative virulence factors, and six putative antibiotic resistance genes were found using CARD (https://card.mcmaster.ca/analyze/rgi, RGI_6.0.3, CARD_3.2.8, perfect/strict/loose, BlastP subject coverage >99% and BlastP identity >96%) (19). It also contained several predicted oxidative stress genes and transcriptional regulators including *perR*, *fur*, catalase, superoxide dismutase, and *ahpC* with potential roles in aerotolerance. The plasmid contained 56 CDS and carried *tetO* (tetracycline resistance gene), *aphA* (aminoglycoside 3′-phosphotransferase), *sat-4* (streptothricin acetyltransferase), *ant(6)-I* (aminoglycoside 6-nucleotidyltransferase), type IV secretion system, and multiple predicted genes for conjugative transfer proteins.

## Data Availability

The genome sequences of the chromosome and plasmid for this isolate are available in the NCBI Database within Bioproject: PRJNA1026992, Genbank accession numbers CP136638 (*Campylobacter jejuni* S2_20 chromosome) and CP136639 (*Campylobacter jejuni* S2_20 plasmid pCJABK49), respectively. Raw reads are available in the SRA database with SRA accession numbers SRX22060912 (Illumina reads) and SRX22060913 (ONT long reads).
